# Genome-wide analysis of HSF family and overexpression of *PsnHSF21* confers salt tolerance in *Populus simonii × P. nigra*

**DOI:** 10.3389/fpls.2023.1160102

**Published:** 2023-04-26

**Authors:** Qing Guo, Ran Wei, Min Xu, Wenjing Yao, Jiahui Jiang, Xujun Ma, Guanzheng Qu, Tingbo Jiang

**Affiliations:** ^1^ State Key Laboratory of Tree Genetics and Breeding, Northeast Forestry University, Harbin, China; ^2^ School of Architecture and Civil Engineer, Heilongjiang University of Science and Technology, Harbin, China; ^3^ Co-Innovation Center for Sustainable Forestry in Southern China/Bamboo Research Institute, Nanjing Forestry University, Nanjing, China

**Keywords:** *Populus simonii × P. nigra*, heat shock transcription factor, salt stress, PsnHSF21, transcriptional regulation

## Abstract

Heat shock transcription factor (HSF) is an important TF that performs a dominant role in plant growth, development, and stress response network. In this study, we identified a total of 30 HSF members from poplar, which are unevenly distributed on 17 chromosomes. The poplar HSF family can be divided into three subfamilies, and the members of the same subfamily share relatively conserved domains and motifs. HSF family members are acidic and hydrophilic proteins that are located in the nucleus and mainly carry out gene expansion through segmental replication. In addition, they have rich collinearity across plant species. Based on RNA-Seq analysis, we explored the expression pattern of *PtHSFs* under salt stress. Subsequently, we cloned the significantly upregulated *PtHSF21* gene and transformed it into *Populus simonii* × *P. nigra*. Under salt stress, the transgenic poplar overexpressing *PtHSF21* had a better growth state and higher reactive oxygen scavenging ability. A yeast one-hybrid experiment indicated PtHSF21 could improve salt tolerance by specifically binding to the anti-stress cis-acting element HSE. This study comprehensively profiled the fundamental information of poplar HSF family members and their responses to salt stress and specifically verified the biological function of *PtHSF21*, which provides clues for understanding the molecular mechanism of poplar HSF members in response to salt stress.

## 1 Introduction

Plants are frequently affected by adverse environments in growth and development, such as high temperature, salt injury, drought, cold, and other abiotic threats, insect pests, viruses, and other biotic stresses (Huang et al., 2021). Nowadays, saline-alkali hazards have become universal problems worldwide (Polle and Chen, 2015). Soil salinization can change soil properties, reduce soil water potential, and lead to soil consolidation, which restricts plant growth and development (Evelin et al., 2019). As sessile organisms, plants cannot actively escape adverse environments due to their own fixed nature, so they rely on physiological and biochemical mechanisms to survive under stress (Shao et al., 2007; Abdullah et al., 2018). Therefore, plants have evolved a series of complex and effective strategies to maintain normal physiological metabolism and stress-resistant growth (Zhou et al., 2009). Transcription factors (TF) are sequence-specific DNA-binding protein molecules that can regulate downstream gene expression by binding to specific cis-acting elements in promoter regions (Shao et al., 2015). Increasing reports have indicated that many kinds of TF carry out a significant role in plant stress resistance. For example, heat shock factor (HSF) can activate transcriptional responses to salinity and oxidative defense in *Populus euphratica* (Shen et al., 2013). MYB-related TF is widely involved in the phosphate starvation response and the tolerance to extreme cold, drought, and salt stress (Sun et al., 2018). The *PsnHDZ63* gene can improve salt tolerance of poplar (Guo et al., 2021a). ABRE binding factor and MYC are directly involved in the signaling pathways of abscisic acid (ABA) and jasmonic acid (JA) (Yoon et al., 2020). WRKY proteins recognize and activate a variety of plant defense genes (Yu et al., 2001).

In particular, HSF is a type of TF that widely exists in eukaryotes and carries out an important role in signal reception and transmission, downstream gene regulation, and stress resistance in plants (Ohama et al., 2017). Highly conserved HSF protein is composed of five basic functional domains: DNA binding domain (DBD), adjacent dimer oligomeric domain (OD), nuclear localization signal (NLS), nuclear export signal (NES), and C-terminal activating peptide protein (CTD) (Li et al., 2014; Guo et al., 2016). DBD is the most conserved domain in HSF, which forms a compact sphere consisting of three α-helical bundles and four inversely parallel β-folded layers. DBD ensures that HSF members specifically bind to cis-acting elements in the target gene promoter, therefore regulating target gene transcription (Sangster and Queitsch, 2005). OD consists of two hydrophobic heptapeptide repeat regions, HR-A and HR-B. According to the differences between HR-A and HR-B, HSF is divided into three subfamilies (HSFA, HSFB, and HSFC) (Nover et al., 2001; Baniwal et al., 2004). NLS is composed of basic amino acids, and NES is rich in leucine (Lyck et al., 1997; Heerklotz et al., 2001). CTD is the least conserved region, which consists of AHA motifs and exhibits the characteristics of transcriptional activation. The first plant HSF gene was cloned from a tomato in 1990. So far, more and more HSFs have been found in many species. For instance, 21 HSF members were identified in *Arabidopsis thaliana*, 25 in rice, 27 in potato, 82 in wheat, 25 in corn, and 19 in grape (Wang et al., 2009; Lin et al., 2011; Guo et al., 2016; Tang et al., 2016; Yang et al., 2016; Duan et al., 2019). Previous studies on individual HSF subfamilies have shown that HSF members are widely involved in plant development and stress responses. Overexpression of *HsfA1b* could significantly increase plant yield, harvest index, and plant resistance to pathogens (Bechtold et al., 2013). AtHSFA7b regulates the expression of the target gene by combining with E-box-like elements to mediate a series of physiological changes, including maintaining cell ion homeostasis, lowering water loss rate, reducing reactive oxygen species accumulation, and regulating osmotic pressure, to improve plant salt tolerance (Zang et al., 2019). Overexpression of the *AtHsfA1b* and *AtHsfA1d* genes can enhance drought and heat tolerance in tomatoes (Higashi et al., 2013). In addition, heterologous overexpression of *BcHsfA1* or *HmHsp70* genes in tobacco can enhance heat tolerance (Zhu et al., 2018; Xu et al., 2020). Overexpression of the *HsfA1* gene from *Lilium brownii* can upregulate the heat shock and betaine synthase genes in *A. thaliana* (Gong et al., 2014). *HsfA1d* transgenic pea decreased the content of H_2_O_2_ under heat stress and significantly increased the activity of antioxidant enzymes (Shah et al., 2020).

As perennial woody plants, poplar has experienced various environmental pressures and seasonal changes. The genome of *Populus trichocarpa* was first published in 2006, which makes it an ideal genetic model for forest genetics and breeding (Tuskan et al., 2006). *Populus simonii* × *Populus nigra* is a hybrid of *P. simonii* and *P. nigra*, which has the advantages of rapid growth, excellent quality, easy survival of cutting, strong adaptability and high development potential. However, salinized soil seriously restricts the growth range of *P. simonii* × *P. nigra* (Guo et al., 2021a). How to improve the salt tolerance of woody plants has become an important research topic. In this study, we identified 30 HSF members from poplar and analyzed their phylogeny, gene structure, conserved domain, promoter cis-acting elements, chromosome location, collinearity, and evolution pattern. In addition, we profiled their expression pattern under salt stress by RNA-Seq and screened out a highly salt-induced *PsnHSF21* gene. We confirmed it was targeted to the nucleus and had self-activating activity, and it can specifically bind to the HSE element. Moreover, we obtained transgenic *P. simonii* × *P. nigra* over-expressing *PsnHSF21* and verified the transgenic poplar displayed growth and physiological advantages under high salt stress. These results are helpful to understand the mechanism of salt resistance and the genetic improvement of HSF members in poplar.

## 2 Materials and methods

### 2.1 Identification of poplar HSF family

The genome information of poplar HSF family genes and their conserved domains was obtained from Phytozome (https://phytozome-next.jgi.doe.gov/). The hidden Markov model (HMM) (Evalue <1 × 10^−5^) of HSF (PF00447) was downloaded from the Pfam database (https://pfam.xfam.org/) (Finn et al., 2011). Poplar HSF sequences were obtained by BLASTP-HMMER, and sequence accuracy was further verified by Pfam and NCBI-CDD (https://www.ncbi.nlm.nih.gov/cdd) (Finn et al., 2016). Multiple sequence alignment was carried out through CLUSTALX 2.0 (Zhao et al., 2020) and visualized with DNAMAN and Web Logo (Guo et al., 2021b). Subcellular localization was predicted by PSORT (http://psort1.hgc.jp/form.html) with default parameters. Physical and chemical parameters were predicted by ExPASy (https://web.expasy.org/protparam/), including molecular weight (MW), isoelectric point (PI), instability index (II), and the average hydrophilic coefficient of proteins (Huang and Xu, 2008).

### 2.2 Phylogeny and sequence analysis

The sequences of 24 HSF members of *A. thaliana* were downloaded from the TAIR database (https://www.arabidopsis.org/). Multiple-sequence alignment was conducted by CLUSTALX 2.0. A phylogenetic tree was constructed by the NJ method in MEGA7.0, and the reliability of branches was evaluated with 1,000 Bootstrap repeated samplings (Kumar et al., 2016). Motif identification was predicted by MEME (https://meme.suite.org/meme/info/status.Service=MEME&id=app.MEME5.5.016727959406951083280962) with the maximum motif number of 10 (Bailey et al., 2009). The conserved domain was analyzed by the NCBI-CD-Search program (https://www.ncbi.nlm.nih.gov/Structure/cdd/wrpsb.cgi). The information about UTR, intron, and CDS was obtained from the GFF annotation of the poplar genome and visualized by TBtools (Chen et al., 2020).

### 2.3 Genetic evolution of the poplar HSF family

Genome information for *A. thaliana* and *Oryza sativa* was obtained from the Ensembl Plants database (http://plants.ensembl.org/index.html). Tandem repeatability of the poplar HSF gene and gene collinear relationships across plant species were identified by BLASTP and the multiple collinear scanning tool (MCScanX) (Wang et al., 2012). The chromosome location of the poplar HSF gene was shown by TBtools (Tuskan et al., 2006). Ka/Ks replacement rate of homologous HSF genes and evolution time T (T = Ks/2λ, λ = 9.1 × 10^−9^) were also calculated by TBtools (Chen et al., 2020).

### 2.4 Analysis of cis-acting elements in the promoter

The PlantCARE database (http://bioinformatics.psb.ugent.be/webtools/plantcare/html/) was used to predict the cis-acting elements in the upstream 2,000 bp promoter sequences of poplar HSF genes. Furthermore, the results were visualized by TBtools (Chen et al., 2020).

### 2.5 Expression analysis of poplar HSF family genes

*P. simonii* × *P. nigra* seedlings from the same poplar clone at Northeast Forestry University were cultured in water under the light/dark cycle of 16/8 h and 26 °C/22 °C, until new roots and new leaves sprouted (Yao et al., 2019). A total of 60 hydroponic seedlings with similar growth states were selected for salt treatment: 30 for 150 mM NaCl solution and 30 for hydroponic control. After 24 h, the roots, stems, and leaves were collected and preserved in liquid nitrogen, then transported to GENEWIZ Company (www.GENEWIZ.com) for RNA-Seq on the IIIumina HiSeq 2500 platform. The analysis of differentially expressed genes (DEGs) was carried out by the DESeq method in R software, and the standards of significant DEGs include |log2 fold change (FC)| ≥1 and a p-value ≤0.05. A Venn diagram was drawn by Venny2.1.0 (https://bioinfogp.cnb.csic.es/tools/venny/index.html) (Anders and Huber, 2010). The relative expression levels of significant differentially expressed *PtHSFs* screened by RNA-Seq were verified by RT-qPCR. The internal reference was *Actin*, and the primers of *PtHSFs* for RT-qPCR are listed in **Supplementary Table S1**. The relative expression level in transgenic poplar was calculated by the 2^−ΔΔ^ Ct method as previously described (Guo et al., 2021b). Furthermore, the LSD test (P <0.05) of SPSS software was performed to examine the significance of gene expression levels (Zhao et al., 2021).

### 2.6 Cloning and sequence analysis of the P*snHSF21* gene

Total RNA was extracted using the TaKaRa MiniBEST Plant RNA Extraction Kit (Takara, Dalian), and cDNA was synthesized using the PrimeScript™ reverse RT reagent kit (Takara, Dalian). The cDNA fragment of the *PtHSF21* gene was cloned by PCR with specific primers (**Supplementary Table S2**) (Goodstein et al., 2012), and it was sequenced by Sangon (http://www.sangon.com/). The sequence was compared by Blastn (http://blast.ncbi.nlm.nih.gov/Blast.cgi). The conserved domain of PtHSF21 was analyzed by the NCBI-CD-Search program (https://www.ncbi.nlm.nih.gov/Structure/cdd/wrpsb.cgi), and the protein structure of PtHSF21 was predicted by Swiss Model (https://swissmodel.expasy.org/interactive/NNZZXxy/model/) (Zhang et al., 2022). The homologous proteins of PtHSF21 were screened by NCBI-blast, and their phylogenetic tree was represented by the NJ method in MEGA7.0, with the reliability of branches being evaluated with 1,000 Bootstrap repeated samplings (Kumar et al., 2016). The potential proteins interacting with PtHSF21 were predicted by String (https://cn.string-db.org/).

### 2.7 Subcellular localization analysis of PsnHSF21 protein

The coding region sequence of *PsnHSF21* without a stopping codon was introduced into the pBI121-GFP vector by SalI and SpeI restriction sites. The PCR primers can be found in **Supplementary Table S3**. The fusion vectors pBI121-PsnHSF21-GFP and pBI121-GFP as controls were transferred into *Agrobacterium tumefaciens* GV3101, respectively. Moreover, the Agrobacterium solutions were injected into the lower epidermis of the strong leaves, which belong to one-month-old tobacco soil seedlings after transplanting. The injected leaves were cultured in the dark for 24 h and then observed for a green fluorescence signal under a Zeiss laser confocal microscope (ZEISS LSM 800).

### 2.8 Generation of transgenic poplar overexpressing *PsnHSF21*

The tissue culture poplar seedlings were used for gene transformation by the leaf disk transformation method. The leaves were immersed in solutions of GV3101 containing pBI121-PsnHSF21-GFP for 10 min and cultured in the dark for 2–3 days. Then the leaves were transferred to a differentiation medium composed of WPM, 0.1 mg/L NAA, 0.2 mg/L 6-BA, 0.5 mg/L Gas, and 50 mg/L Kan to induce adventitious buds. The 2-cm buds can be transferred to 1/2 MS rooting medium with 50 mg/L Kan. The Kan-resistant seedlings were confirmed by molecular detection with WT DNA as a negative control and plasmid as a positive control. In addition, the relative expression levels of *PsnHSF21* in transgenic poplar were calculated by RT-qPCR with three biological repeats per sample.

### 2.9 Salt tolerance of transgenic poplar overexpressing *PsnHSF21*

Two-month-old transgenic poplars (OE1, OE2, OE3, and OE4) and WT plants as controls were irrigated with 200 mM NaCl solution for 7 days. The growth indexes, such as fresh weight, plant height, main root length, and lateral root number, were measured. Furthermore, leaves in three to four layers were collected for the determination of physiological indexes, including POD, proline, and MDA, using the Nanjing Jiancheng Bioengineering Research Institute kit. The relative expression levels of stress resistance genes such as SOD, POD, HRG, ABA, and GA were analyzed by RT-qPCR. The relevant primers can be found in **Supplementary Table S4**. Histochemical staining of DAB and Evans blue was used to predict the activities of hydrogen peroxide and superoxide in plants. Tissue culture seedlings at one month old were soaked in 200 mM NaCl for 24 h, and the leaves were immersed in DAB and Evans blue solution in the dark for 24 h and then decolorized with ethanol (Sekulska-Nalewajko et al., 2016).

### 2.10 Self-activating activity and HSE cis-element interaction of the PsnHSF21 protein

The CDS sequence of the PsnHSF21 gene was fused into pGBKT7 to form pGBKT7-PsnHSF21 at two restriction sites, NdeI and SmaI. The related primers can be found in **Supplementary Table S5**. The fusion vectors pGBKT7-PsnHSF21, pGBKT7 (negative control), and pGBKT7-53/pGADT7-T (positive control) were respectively transferred into the Y2H Gold yeast strain according to the Yeast Maker yeast transformation system and then inoculated on SD/-Trp and SD/-Trp/-His/X-α-Gal solid medium to detect the self-activating activity of the PsnHSF21 protein.

The stress-resistant element HSE (GAATTC) was refolded in series three times and then inserted into the pAbAi vector to form the bait vector AB-HSE, which was prepared for AB-HSE-Y1H competent cells. The CDS sequence of the PsnHSF21 gene was inserted into pGADT7 to form the bait vector AD-PsnHSF21 by restriction sites NdeI and BamHI. The primers are listed in **Supplementary Table S5**. The AD-PsnHSF21 vector was transferred into yeast cells and cultured on SD/-Leu/AbA (200 ng/ml) for 3–5 days. The positive clones were diluted and presented in culture medium.

## 3 Results

### 3.1 Identification of poplar HSF family members

**Figure 1:**
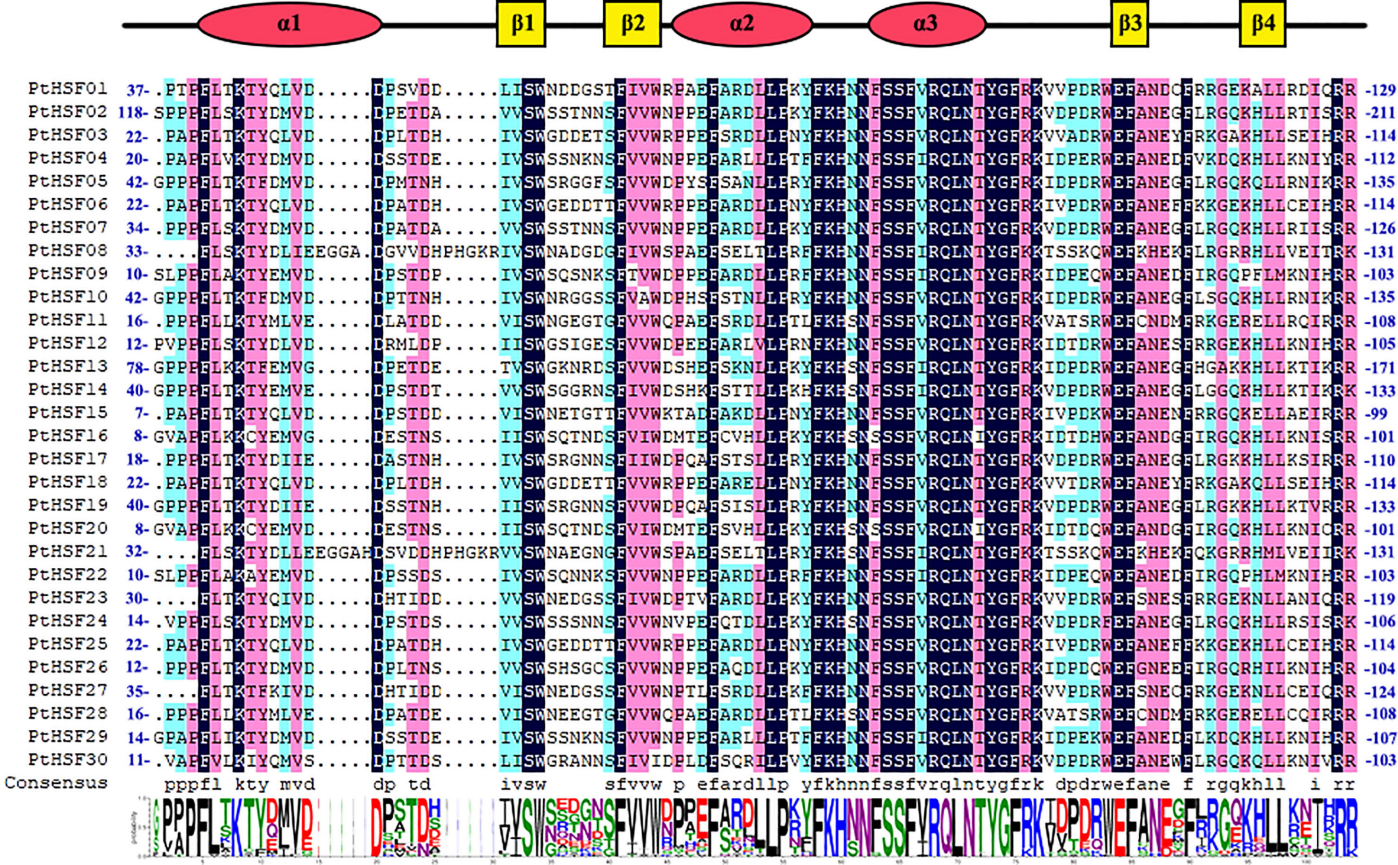
Comparison of the HSF domain of PtHSFs. Secondary structure elements of the DBDs (α1-β1-β2-α2-α3-β3-β4) based on JNet structure predictions are shown above the alignment; α-helices are indicated by red circular legends, and β-folds are indicated by yellow square legends. The total height of each column of letters in the Web logo section indicates the conservation of each position, and the height of each letter represents the relative rating of the corresponding amino acids.

Allowing the hidden Markov model (HMM) of HSF (PF00447) as a clue, we searched the poplar genome and identified a total of 30 HSF proteins by Pfam and NCBI-CDD multiple verification. The repetitive regions of the 30 PtHSF proteins were extracted for multiple sequence alignment and visualization (**Figure 1**). The DBD domains of PtHSF proteins include three α-helical bundles and four inversely parallel β-folded layers. PtHSF proteins have 210–596 aa, with a protein molecular weight of 24,028.43–65,369.32 Da. The isoelectric points of the 30 PtHSF proteins are different. The isoelectric points of PtHSF06, PtHSF08, PtHSF11, PtHSF21, PtHSF25, and PtHSF30 are more than 7; these are basic proteins, of which PtHSF21 is the largest with 9.35. The values of the other 24 PtHSFs are all less than 7 and are acidic proteins, of which PtHSF20 is the smallest with 4.7. The instability index of PtHSF proteins varies greatly, in which the value of PtHSF15 and PtHSF16 is low and their stability is poor. PtHSFs are all hydrophilic proteins, of which PtHSF10 has the weakest hydrophilicity (−0.914) and PtHSF06 has the strongest hydrophilicity (−0.468). Based on the prediction of subcellular localization, all poplar HSF family proteins are in the nucleus, except PtHSF16, which is also found in the cytoplasm (**Table 1**).

**Table 1:** Analysis of *PtHSF* genes.

Gene Name	Accession number	AA	Molecular Weight	Theoretical pI	Instability Index	Grand Average of Hydropathicity	Subcellular Location	AtSBP Ortholog
*PtHSF1*	*Potri.001G108100.1*	344	36,826.62	4.97	55.66	−0.559	Nuclear	*AT4G11660.1*
*PtHSF2*	*Potri.001G138900.1*	596	65,369.32	5.49	64.08	−0.532	Nuclear	*AT1G32330.1*
*PtHSF3*	*Potri.001G273700.1*	271	31,292.34	6.63	56.72	−0.61	Nuclear	*AT1G46264.1*
*PtHSF4*	*Potri.001G320900.1*	491	54,702.69	5.77	56.61	−0.77	Nuclear	*AT4G13980.1*
*PtHSF5*	*Potri.002G048200.1*	360	41,148.32	5.36	57.79	−0.808	Nuclear	*AT3G22830.1*
*PtHSF6*	*Potri.002G124800.1*	365	40,440.67	8.15	51.74	−0.468	Nuclear	*AT1G46264.1*
*PtHSF7*	*Potri.003G095000.1*	508	55,694.91	4.84	58.55	−0.601	Nuclear	*AT1G32330.1*
*PtHSF8*	*Potri.004G042600.1*	210	24,028.43	9.16	57.4	−0.655	Nuclear	*AT1G32330.1*
*PtHSF9*	*Potri.004G062300.1*	408	46,642.09	5.14	57.47	−0.843	Nuclear	*AT4G18880.1*
*PtHSF10*	*Potri.005G214800.1*	360	40,692.42	5.5	66.48	−0.914	Nuclear	*AT3G22830.1*
*PtHSF11*	*Potri.006G049200.1*	227	26,377.04	8.75	51.8	−0.753	Nuclear	*AT2G41690.1*
*PtHSF12*	*Potri.006G115700.1*	445	49,880.96	5.09	61.8	−0.634	Nuclear	*AT5G03720.1*
*PtHSF13*	*Potri.006G148200.1*	431	48,496.82	5.47	51.38	−0.626	Nuclear	*AT2G26150.1*
*PtHSF14*	*Potri.006G226800.1*	389	43,856.92	4.96	56.8	−0.592	Nuclear	*AT2G26150.1*
*PtHSF15*	*Potri.007G043800.1*	286	31,018.89	4.81	37.61	−0.853	Nuclear	*AT4G36990.1*
*PtHSF16*	*Potri.008G136800.2*	394	44,808.01	4.8	40.54	−0.694	Nuclear, Cytoplasmic	*AT1G67970.1*
*PtHSF17*	*Potri.008G157600.1*	349	40,084.03	5.15	57.84	−0.692	Nuclear	*AT3G22830.1*
*PtHSF18*	*Potri.009G068000.1*	273	31,530.7	6.67	57.32	−0.586	Nuclear	*AT1G46264.1*
*PtHSF19*	*Potri.010G082000.1*	359	41,335.34	5.16	55.43	−0.788	Nuclear	*AT3G22830.1*
*PtHSF20*	*Potri.010G104300.1*	393	44,690.88	4.7	43.14	−0.705	Nuclear	*AT1G67970.1*
*PtHSF21*	*Potri.011G051600.1*	212	24,383.6	9.35	53.41	−0.772	Nuclear	*AT5G16820.2*
*PtHSF22*	*Potri.011G071700.1*	407	46,266.63	5.23	54.95	−0.793	Nuclear	*AT4G18880.1*
*PtHSF23*	*Potri.012G138900.1*	302	33,366.24	5.04	50.83	−0.667	Nuclear	*AT5G62020.1*
*PtHSF24*	*Potri.013G079800.1*	500	55,091.51	5.54	57.66	−0.619	Nuclear	*AT5G16820.2*
*PtHSF25*	*Potri.014G027100.1*	369	41,038.16	8.16	52.12	−0.564	Nuclear	*AT1G46264.1*
*PtHSF26*	*Potri.014G141400.1*	444	50,781.7	5.82	62.5	−0.806	Nuclear	*AT5G45710.1*
*PtHSF27*	*Potri.015G141100.1*	287	31,751.76	5.14	50.8	−0.47	Nuclear	*AT5G62020.1*
*PtHSF28*	*Potri.016G056500.1*	229	26,485.98	6.78	55.59	−0.732	Nuclear	*AT2G41690.1*
*PtHSF29*	*Potri.017G059600.1*	486	54,386.35	5.68	57.9	−0.752	Nuclear	*AT4G13980.1*
*PtHSF30*	*Potri.T137400.1*	259	29,986.36	8.98	49.32	−0.548	Nuclear	*AT3G24520.1*

### 3.2 Phylogenetic tree and gene structure analysis

**Figure 2:**
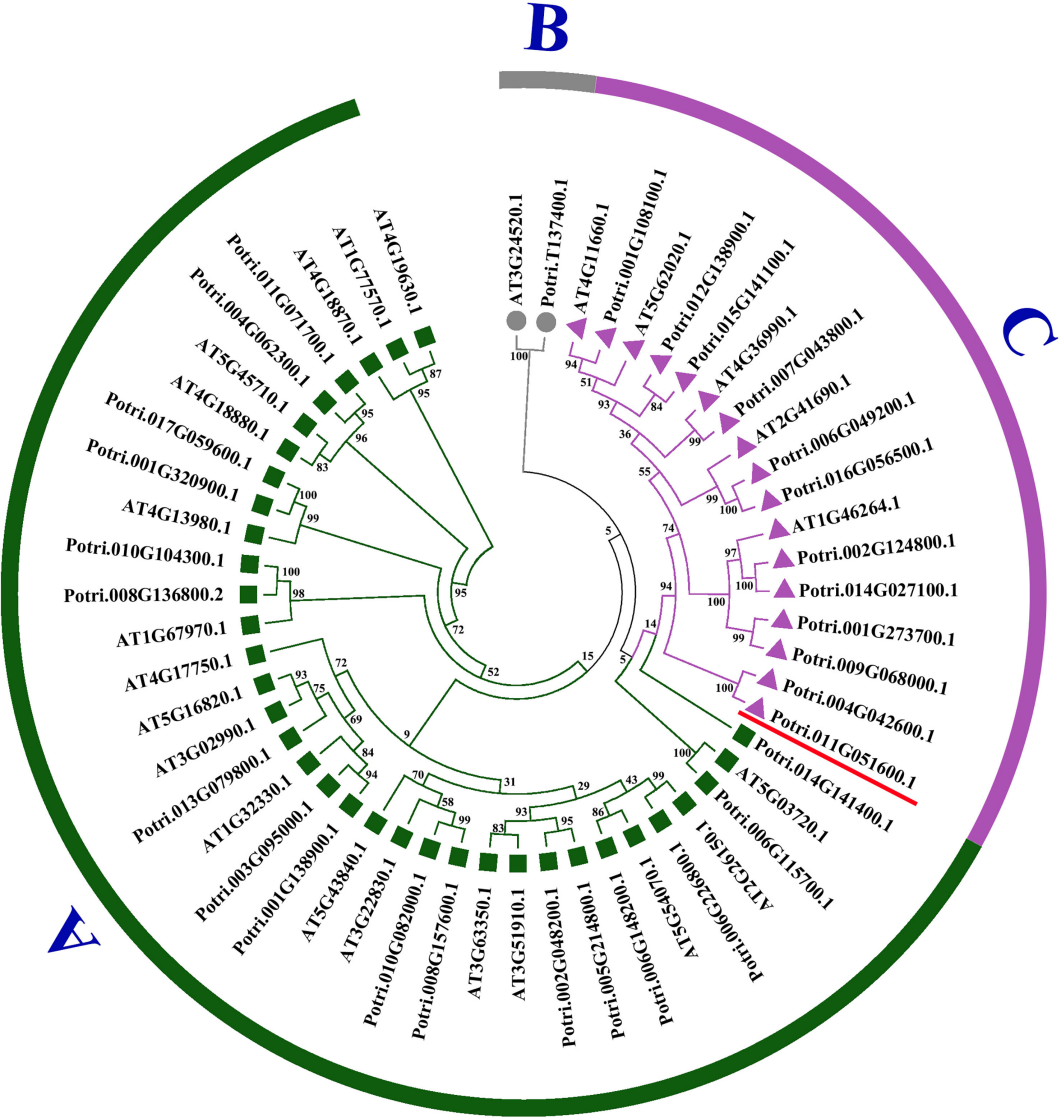
Phylogenetic tree of PtHSFs and AtHSFs. The rootless phylogenetic tree of the HSF protein sequence of poplar and *Arabidopsis thaliana* was drawn by the NJ neighbor method in MEGA7.0. The phylogenetic tree was divided into three groups: **(A–C)**, with each color remaining in one group. Potri.011G051600.1 (PtHSF21) was marked with a red “–.”.

In the study, 30 poplar HSF proteins and 24 HSF proteins from *A. thaliana* were used for constructing a phylogenetic tree (**Figure 2**). The similarity between PtHSFs and AtHSFs is 70%–100%. HSF proteins are divided into three subfamilies, including Classes A, B, and C. Among them, Class A is the largest with 17 PtHSF proteins; Class B is the smallest, which merely has PtHSF06 (Potri.T137400.1); and Class C accounts for 40% of poplar HSF members.

**Figure 3:**
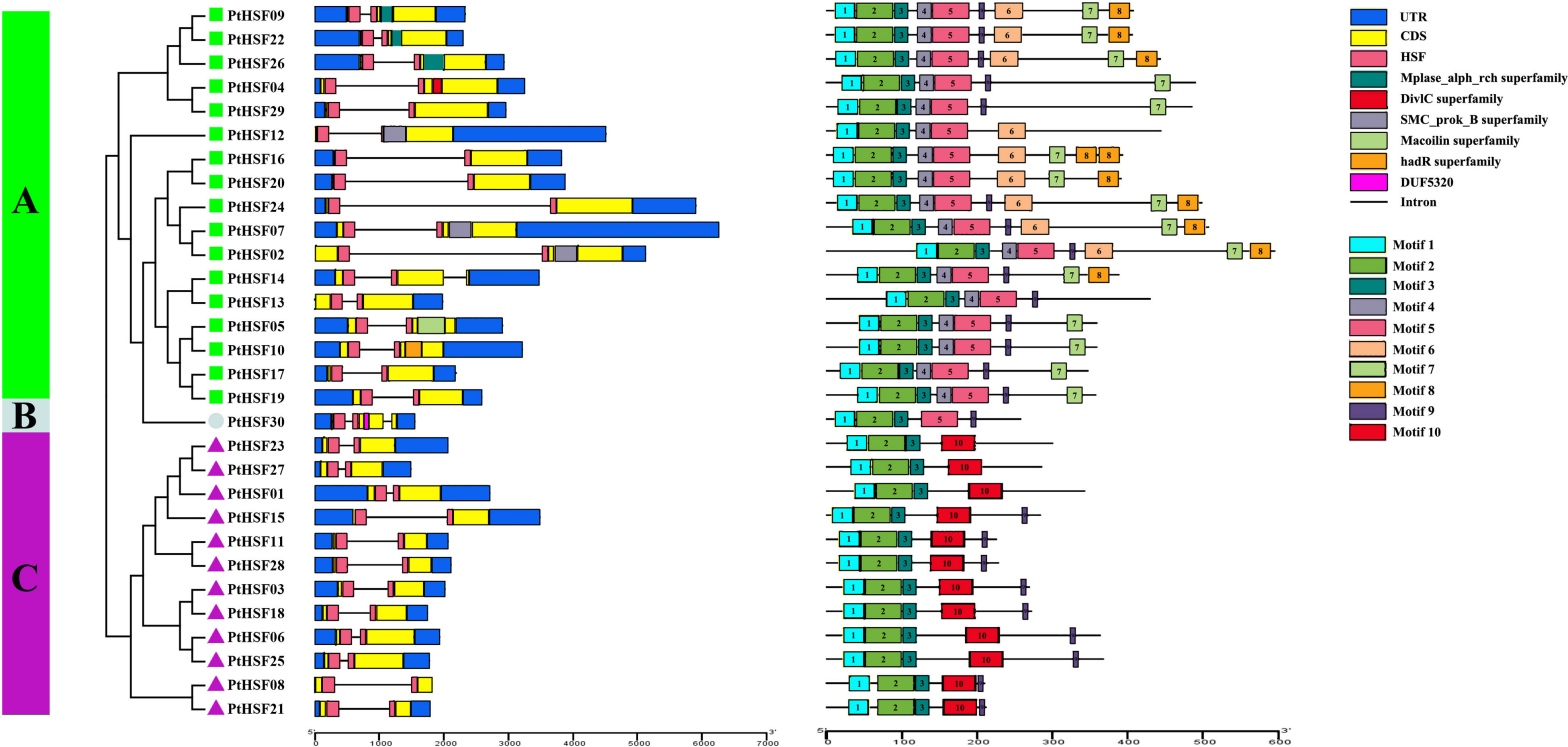
Analysis of HSF gene structure and conserved motif of poplar. The phylogenetic tree was constructed based on 30 HSF protein sequences from poplar, which were divided into three subfamilies. In the gene structure analysis, blue represents the UTR, yellow represents the CDS, pink represents the HSF region, and “–” represents the intron. The conservative motif of the HSF gene is predicted by the MEME website. The number in the color block (1–10) represents motifs 1–10, respectively, and the length of the color block indicates the size of the motif.

As shown in the gene structure map, all PtHSF members have a typical HSF conserved domain, while there are differences in the UTR, CDS, domain, and intron structures among PtHSFs (**Figure 3**). Broadly speaking, the PtHSF members in the same subfamily have similar gene structures except for the HSF domain, Classes A and B members also contain some additional conservative domains. For example, in Class A, PtHSF04 contains the DivlC domain, PtHSF05 contains the Macoilin domain, PtHSF10 contains the hadR domain, PtHSF09, PtHSF22, and PtHSF26 all contain the Mplase_alph_rch domain, and PtHSF02, PtHSF07, and PtHSF12 all contain the SMC_prok_B domain. PtHSF30 in Class C contains the DUF5320 domain. In addition, PtHSFs contain 1–2 introns; 93% of PtHSFs contain one intron, while PtHSF14 and PtHSF30 contain two introns. MEME prediction results indicated PtHSF proteins contain 4–9 conserved motifs, among which the members in Class A contain the most motifs (6–9) and PtHSF01, PtHSF23, and PtHSF27 in Class C have the least, with only four motifs, including motifs 1, 2, 3, and 10. All members include three conserved motifs, including motifs 1–3. Except for the above three motifs, Class A members contain motifs 4 and 5, and Class C members contain motif 10. The information for motifs 1 to 10 is given in **Supplementary Figure 1**.

### 3.3 Chromosome mapping and gene evolution analysis

**Figure 4:**
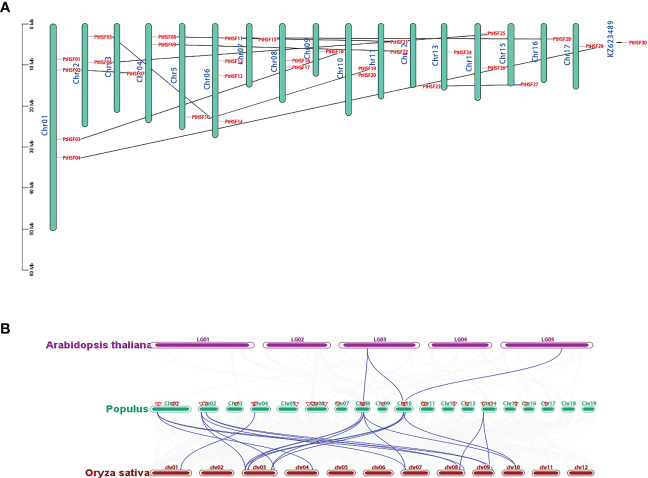
Repetition and collinearity analysis of *PtHSFs* fragments. **(A)** Approximately 30 HSF genes were unevenly distributed on poplar chromosomes, and the segmental repetition relationships were represented by black lines. **(B)** Collinearity analysis of HSF genes in poplar, *Arabidopsis*, and rice. The blue line represents the HSF lineal homologous gene pairs, and the red triangle indicates *PtHSFs.*.

The 30 PtHSF members have chromosomes that are unevenly distributed on poplar chromosomes. It is unusual there exists a *PtHSF30* distributed on the KZ623489 scaffold. Chromosomes 1 and 6 contain four HSF genes; chromosomes 2, 4, 8, 10, and 11 contain two HSF genes; chromosomes 18 and 19 have no HSF genes; and the other chromosomes contain one HSF gene (**Figure 4A**). Phylogenetic analysis indicated that the whole genome duplication of HSF family genes may have occurred in poplar, which were then analyzed by TBtools-McSxanX. There were 11 duplicate pairs in *PtHSFs* (**Figure 4A**), but no tandem repeats appeared, indicating that fragment repetition remains the main factor of gene expansion in the poplar HSF family. To further study the evolutionary constraints of *PtHSFs* genes, we analyzed the non-synonymous substitution rate Ka, synonymous substitution rate Ks, and Ka/Ks values of the 11 homologous *PtHSFs* gene pairs (**Table 2**). The non-synonymous substitution rate Ka indicates that the amino acid changes were caused by base replacement, while the synonymous substitution rate Ks indicates that the amino acid changes did not occur due to base replacement. The synonymous substitution rate Ks is used to predict the occurrence time of genome-wide repetitive events, which are between 0.2397 and 1.9035. So large-scale gene repetition events may have occurred about 104.58887 MAY (million years ago) in poplar. The most recent event happened at 13.16965 MAY (million years ago). Moreover, the Ka/Ks values of *PtHSFs* gene pairs are all less than 1, indicating that poplar HSF family genes have experienced strong purification selection.

**Table 2:** The Ka/Ks values of *PtHSFs* paralogous gene pairs.

No.	Paralogous Genes	Ka	Ks	Ka/Ks	MAY
1	PtHSF02(Chr01)/PtHSF07(Chr03)	0.0736	0.2508	0.2936	13.7814
2	PtHSF03(Chr01)/PtHSF18(Chr09)	0.0550	0.3091	0.1780	16.9833
3	PtHSF04(Chr01)/PtHSF29(Chr17)	0.0598	0.2441	0.2450	13.4143
4	PtHSF05(Chr02)/PtHSF10(Chr05)	0.0647	0.2504	0.2585	13.7571
5	PtHSF19(Chr10)/PtHSF10(Chr05)	0.2654	1.9035	0.1394	104.5889
6	PtHSF21(Chr11)/PtHSF08(Chr04)	0.0983	0.3470	0.2833	19.0642
7	PtHSF22(Chr11)/PtHSF09(Chr04)	0.0630	0.2397	0.2629	13.1696
8	PtHSF23(Chr12)/PtHSF27(Chr15)	0.1241	0.3667	0.3385	20.1472
9	PtHSF25(Chr14)/PtHSF06(Chr02)	0.0218	0.3139	0.0695	17.2490
10	PtHSF28(Chr16)/PtHSF11(Chr06)	0.0370	0.2440	0.1517	13.4067

Ka indicates non-synonymous substitution rate.

Ks indicates synonymous substitution rate.

MYA indicates million years ago.

To further confirm the phylogenetic relationship of HSF family members, the collinear relationships of nine *PtHSFs* from poplar, two *AtHSFs* from *A. thaliana*, and twelve *OsHSFs* from rice were analyzed. The HSF family members constitute 23 lineal homologous gene pairs, including three *PtHSFs*-*AtHSFs* pairs and twenty *PtHSFs*-*OsHSFs* pairs (**Figure 4B**; **Supplementary Table S6**). Ordinarily, *PtHSFs* can correspond to one or two HSF genes from other plant species. Interestingly, *PtHSF19* corresponds to two *AtHSFs* (*AT3G22830.1* and *AT5G43840.1*) and three *OsHSFs* (*LOC_Os03g06630.1*, *LOC_Os03g53340.1*, and *LOC_Os10g28340.1*), indicating that this gene is critically valuable in the evolution of the poplar HSF family.

### 3.4 Cis-acting elements of PtHSF promoters

**Figure 5:**
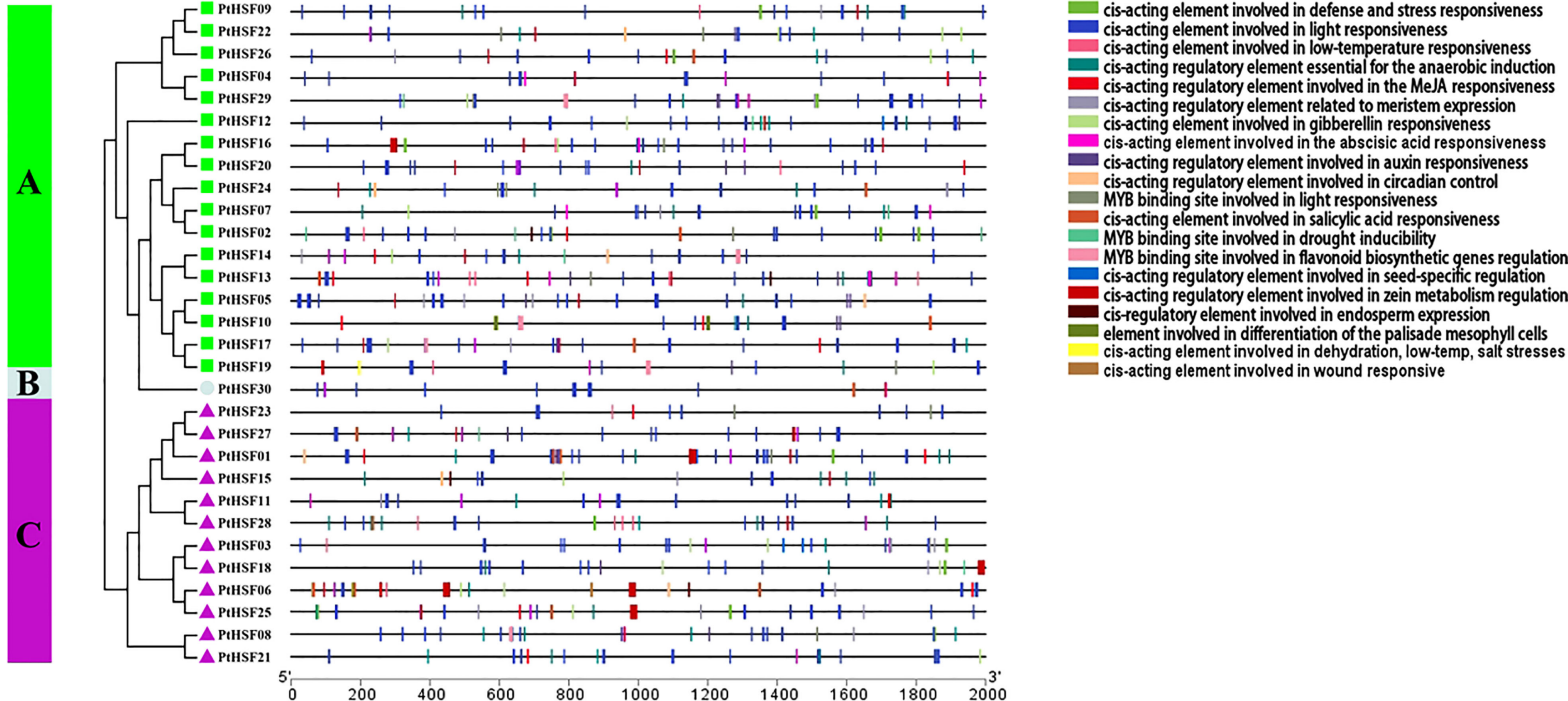
Analysis of Cis-acting elements in the HSF gene promoter in poplar. The upstream 2,000 bp sequences of 30 HSF genes were truncated and analyzed, and the blocks of different colors represented cis-acting elements with diverse functions.

Cis-acting elements are the core part of gene promoters, which carry out a significant regulatory function in gene expression. We analyzed the cis-acting elements in the upstream 2,000 bp promoter sequences of *PtHSFs* by PlantCARE. Among these genes, the *PtHSF01* promoter contains the most cis-acting elements (37), while the *PtHSF23* promoter contains the least cis-acting elements (13) (**Figure 5**). Additionally, these promoters include many elements that regulate development, such as meristem-specific expression regulatory elements, seed-specific expression regulatory elements, endosperm-specific expression regulatory elements, palisade mesophyll cell differentiation regulatory elements, and so on. Many abiotic stress response elements, such as light response elements, low temperature response elements, salt response elements, drought response elements, and wound response elements, and hormone synthesis-related elements, such as jasmonic acid response elements, gibberellin response elements, and auxin response elements, were also present in *PtHSFs* promoters.

### 3.5 Expression pattern of PtHSFs under salt stress

**Figure 6:**
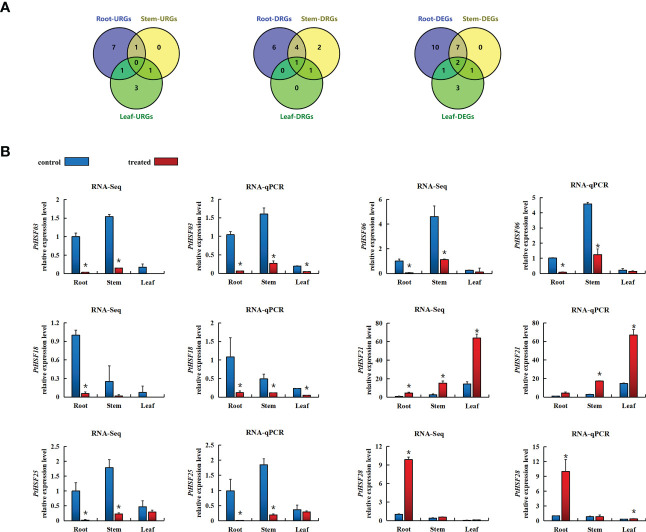
**(A)** VENN diagram of differential gene expression in the roots, stems, and leaves of poplar after salt stress. The figure indicates the number of DEGs in response to salt stress in different tissues, upregulating the number of DEGs and downregulating the number of DEGs. DEG means differentially expressed gene, URG means represent upregulating differentially expressed gene, and DRG means represent downregulating differentially expressed gene. **(B)** After salt stress, the expression pattern of the HSF gene in different poplar tissues was based on RNA-Seq and RT-qPCR analysis. Blue indicates the control group, and red indicates the salt treatment. Based on the expression level of corresponding genes in the roots of the control treatment, the relative expression level of each gene under salt stress was calculated. The error line represents the standard deviation (SD) of biological replication. “*” indicates there is a significant difference (P <0.05).

The expression pattern of *PtHSFs* in the root, stem, and leaf of poplar under salt stress was analyzed by RNA-Seq (**Supplementary Sheet**). In the root, twenty *PtHSFs* responded to salt stress, including nine upregulated genes and eleven downregulated genes. There were ten salt-induced *PtHSFs* in the stem; two were upregulated and eight were downregulated. There were seven *PtHSFs* in the leaf of poplar in response to salt stress, including five upregulated genes and two downregulated genes. Simultaneously, we drew a Venn diagram of the differentially expressed genes in the three tissues. *PtHSF03* and *PtHSF05* were differentially expressed in the three tissues. *PtHSF28* was upregulated in leaf and root, *PtHSF26* was upregulated in stem and root, and *PtHSF21* was upregulated in leaf and stem. *PtHSF03* was downregulated in three tissues; *PtHSF06*, *PtHSF18*, *PtHSF25*, and *PtHSF27* were downregulated, in stem and root; *PtHSF05* was downregulated in the stem and leaf (**Figure 6A**). RT-qPCR of 6 DEGs in different tissues was carried out to check the RNA-Seq results, which proved to be consistent. *PtHSF3*, *PtHSF6*, *PtHSF18*, and *PtHSF25* were all downregulated in all three tissues (**Figure 6B**). It is worth paying attention to that one-third of the genes were upregulated in all three tissues, and the expression of *PtHSF28* changed less, while *PtHSF21* was significantly upregulated in both stems and leaves, which was selected as the key research object in the study.

### 3.6 Bioinformatics analysis of *PsnHSF21*

**Figure 7:**
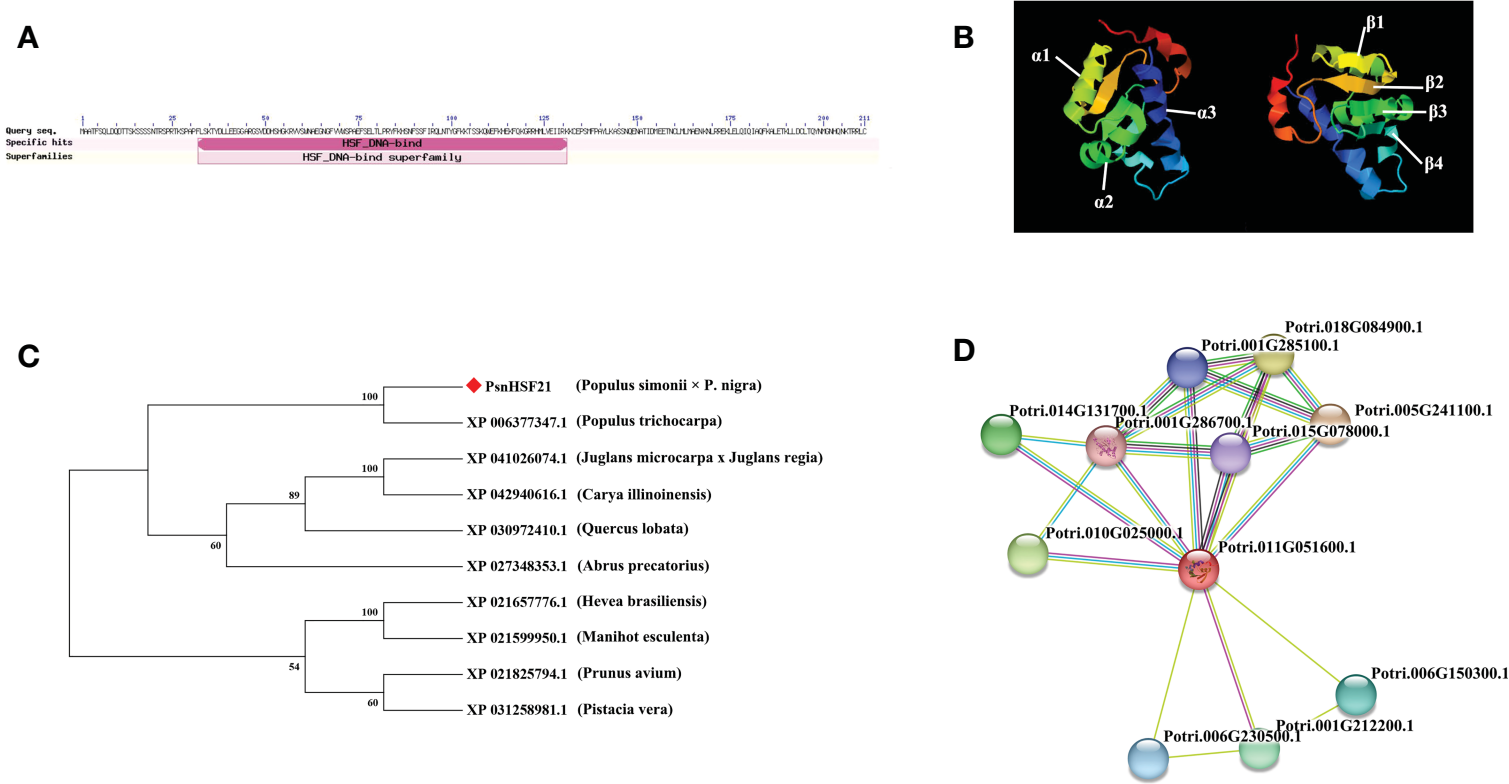
**(A)** Exhibition of the conserved domain of PsnHSF21 protein. **(B)** The schematic diagram of the tertiary structure of the PsnHSF21 protein. **(C)** Phylogenetic relationship between PsnHSF21 and HSFs in various species. **(D)** Prediction of PsnHSF21 interaction protein.

We cloned the *PsnHSF21* CDS with a length of 636 bp from *P. simonii* × *P. nigra*, encoding 212 amino acids with a highly conserved HSF domain (**Figure 7A**), and its protein sequence similarity to PtHSF21 was 96.21%. The protein has three α-helical bundles and four inverted parallel β-folded layers, which is consistent with the protein characteristics of the HSF family (**Figure 7B**). According to the results of NCBI-blast (**Figure 7C**), there were nine proteins highly homologous to PsnHSF21, including HSFs from *P. trichocarpa*, *Hevea brasiliensis*, *Prunus avium*, *Manihot esculenta*, *Juglans microcarpa* × *Juglans regia*, *Abrus precatorius*, *Quercus lobata*, *Pistacia vera*, and *Carya illinoinensis*. Among them, HSF (XP_006377347) from *P. trichocarpa* was most closely related to PsnHSF21, while it was farthest for the HSFs from *P. avium* (XP_021825794.1) and *P. vera* (XP_031258981.1). The prediction results of the protein–protein interaction network (**Figure 7D**) show there were ten proteins interacting with PsnHSF21, namely Potri.001G212200.1, Potri.001G285100.1, Potri.001G286700.1, Potri.005G241100.1, Potri.006G150300.1, Potri.006G230500.1, Potri.010G025000.1, Potri.015G078000.1, Potri.014G131700.1, and Potri.018G084900.1. The average node degree of the network is 4, the local clustering coefficient is 0.791, and the enrichment P value is 0.719.

### 3.7 Subcellular localization of PsnHSF21

**Figure 8:**
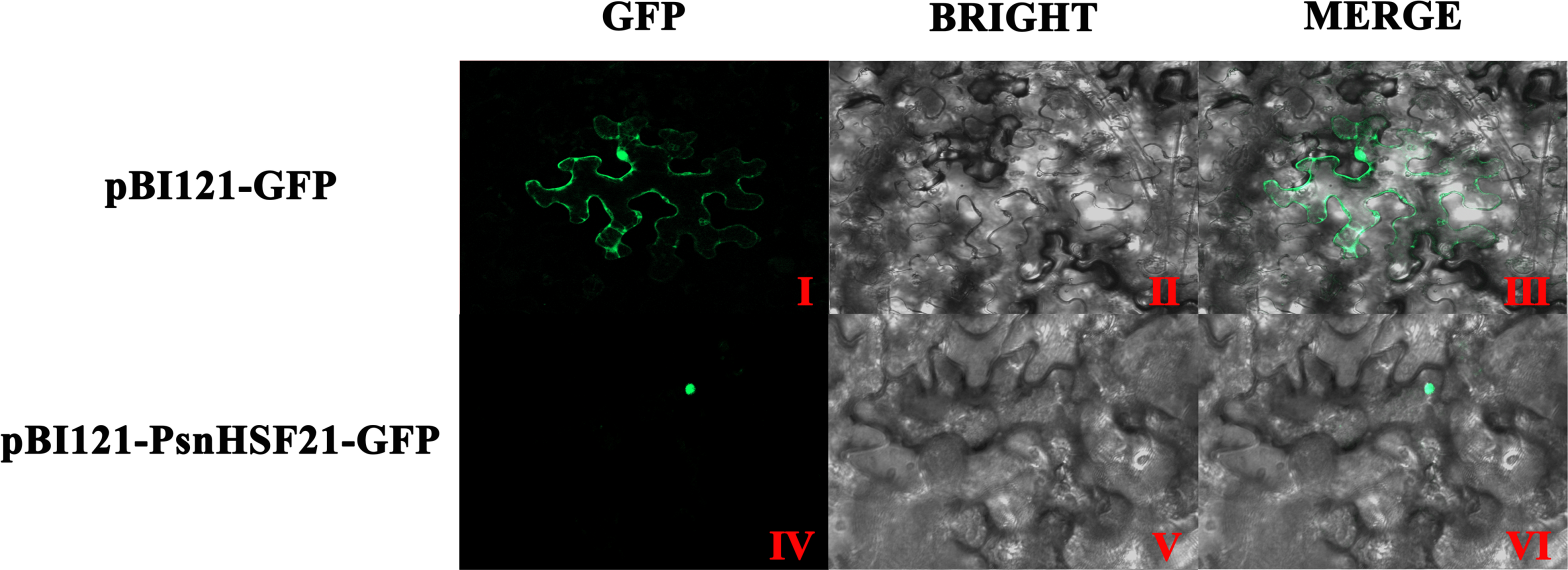
Subcellular localization of PsnHSF21 protein. The pBI121-GFP-PsnHSF21 fusion vector and pBI121-GFP control vector were transferred into tobacco leaf cells by instantaneous transformation. After 24–36 h of transformation, the pictures were imaged by the laser confocal microscope. (I, IV) are GFP fluorescence detection, (II, V) are bright fields, and (III, VI) are superposition fields.

The subcellular localization of PsnHSF21 protein was analyzed in tobacco leaves by transient infection. As shown in **Figure 8**, the green fluorescence signal of pBI121-PsnHSF21-GFP was only observed in the nucleus, whereas it was expressed throughout the cells for the control vector pBI121-GFP, indicating that PsnHSF21 is a nuclear localization protein.

### 3.8 Salt resistance analysis of transgenic poplar overexpressing *PsnHSF21*

**Figure 9:**
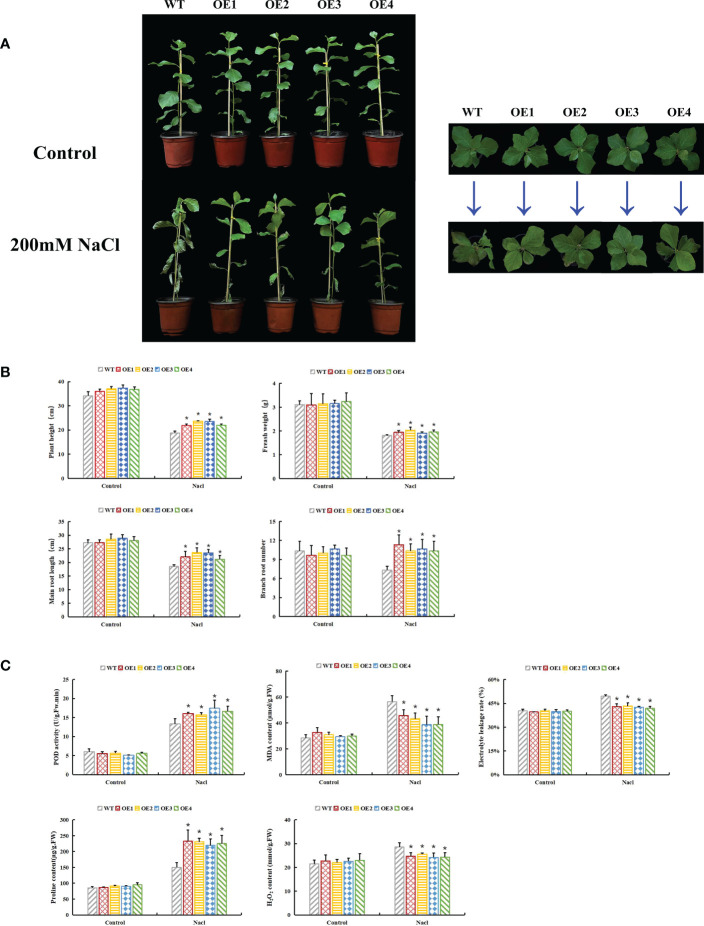
Functional analysis of *PsnHSF21* under salt stress. The seedlings of WT and overexpressed *PsnHSF21* poplar were stressed with water and 200 mM NaCl for 7 days. Control was water and treatment was 200 mM NaCl. **(A)** Display of the growth phenotype. **(B)** The growth index shows plant height, fresh weight, main root length, and lateral root number, three biological repeats. **(C)** Physiological index displays: POD, MDA, proline, electrical conductivity, and H_2_O_2_ content, three biological repeats. The error line represents the standard deviation (SD) of biological replication. “*” indicates there is a significant difference (P <0.05).

Seven transgenic poplar lines overexpressing *PsnHSF21* were obtained by the leaf disc transformation method, which was confirmed by RT-qPCR (**Supplementary Figure 2**). OE1-OE4 with high expression levels were selected for functional analysis. Under normal conditions, there was no obvious morphological difference between WT and OE. Under salt stress, severe salt spots and obvious wilting appeared on the leaves and stems of WT. Compared with WT, the injury degree of transgenic lines was lower (**Figure 9A**). Furthermore, plant height, fresh weight, taproot length, and number of lateral roots of transgenic lines were 17%, 6%, 14%, and 41% higher than those of WT, respectively (**Figure 9B**).

**Figure 10:**
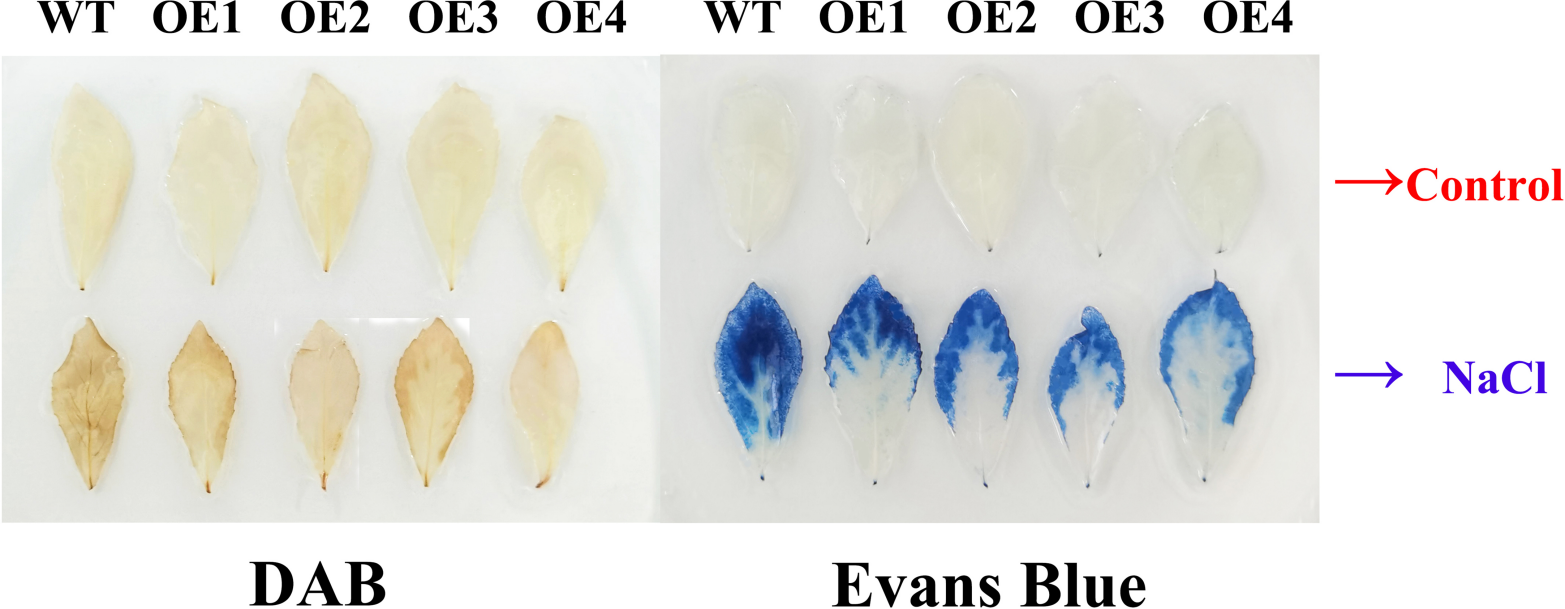
Histochemical staining analysis of poplar leaves. The tissue culture seedling leaves of WT and overexpressed *PsnHSF21* poplar were treated with 150 mM NaCl and water for 24 h, then stained with DAB (diaminobenzidine) and Evans Blue, respectively.

Under salt stress, plants produce a large amount of ROS, and the toxic effect of ROS can inhibit plant development. The activities of peroxidase (POD) and superoxide dismutase (SOD) indirectly reflect the ROS scavenging ability of plants. H_2_O_2_ remains the core component of ROS, which can also oxidize DAB into brown precipitates. Physiological index measurements indicated POD activity, H_2_O_2_ content, MDA content, electrical conductivity, Evans blue, and DAB staining degrees of leaves that displayed no difference between OE and WT under normal conditions. After salt stress, the POD activity of OE was 18%–31% higher than that of WT, and the H_2_O_2_ content of OE was 10%–16% lower than that of WT. MDA content and electrical conductivity of OE decreased by 19%–31% compared with WT. The Evans blue and DAB staining degrees of OE leaves were weaker than those of WT (**Figures 9C**, **10**). Proline is also an important index to measure plant stress resistance. High proline content indicates improved stress tolerance. Under salt stress, the proline content of OE was 1.44–1.55 times higher than that of WT. RT-qPCR analysis showed that the relative expression levels of POD and SOD genes in OE were higher than those in WT (**Supplementary Figure 3**). The results indicated that *PsnHSF21* transgenic poplar lines had stronger scavenging ability for ROS than WT. In addition, the relative expression levels of ABA, GA, and HRG genes associated with stress resistance were higher in OE than those in WT (**Supplementary Figure 3**). The above results showed that overexpression of *PsnHSF21* could improve the salt resistance of transgenic poplars.

### 3.9 Self-activating activity and stress resistant element recognition of PsnHSF21

**Figure 11:**
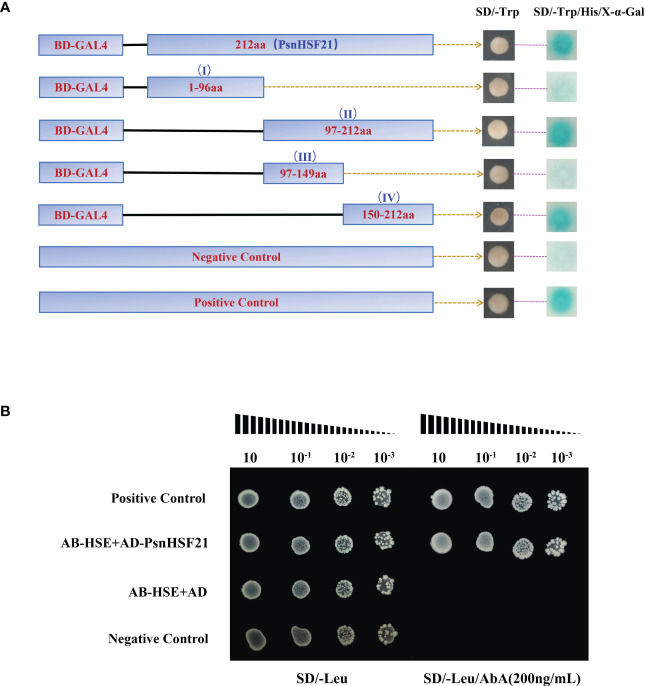
**(A)** Analysis of the PsnHSF21 protein’s self-activating activity. The pGBKT7-PsnHSF21 fusion vector was constructed and transferred into Y2H yeast cells. Transformed cell dots were plated on both SD/-Trp and SD/-Trp/-His/X-α-Gal medium. BD indicates pGBKT7. pGBKT7 was loaded empty as a negative control and pGBKT7-53/pGADT7-T as a positive control. **(B)** Analysis of resistant cis-acting elements identified by PsnHSF21. PsnHSF21 can be specifically bound to HSE (GAATTC) in yeast one-hybrid assays. AB-HSE indicates pAbAi-HSE, AD indicates pGADT7, and AD-PsnHSF21 indicates pGADT7-PsnHSF21 Positive conversion was determined after being diluted with positive yeast on a Leu plate (200 ng/ml) supplemented with AbA (aureobasidin A). The pAbAi-p53/pGADT7-p53 served as positive controls, and the pAbAi-53/pGADT7 served as negative controls.

To identify the transcriptional activity of PsnHSF21, we performed a Y2H assay. It was found that BD-PsnHSF21 could grow normally on both media and turn blue on SD/-Trp/-His/X-α-Gal, indicating it had self-activating activity and could be efficiently transcribed (**Figure 11A**). We divided PsnHSF21 into four segments, including BD-I (1–96 aa), BD-II (97–212 aa), BD-III (97–149 aa), and BD-IV (150–212 aa). The vectors contain four differential segments, which were respectively transformed into Y2H cells to determine the self-activating region of PsnHSF21. As shown in **Figure 11A**, all fragments can survive on SD/-Trp, but only BD-II and BD-IV can turn blue on SD/-Trp/-His/X-α-Gal. Therefore, the self-activated region of the PsnHSF21 protein is located at the C-terminal 150–212 aa, and the amino acid residues in this region are essential for PsnHSF21 activity.

Many studies have proved that the stress-resistant element HSE can bind to HSF family TFs in particular. Therefore, we carried out a yeast one-hybrid experiment with PsnHSF21. The results showed that all combinations could grow normally on SD/-Leu, but only AD-PsnHSF21/AB-HSE and the positive control could grow normally on SD/-Leu/AbA (200 ng/ml) (**Figure 11B**). Therefore, PsnHSF21 may regulate the expression of downstream stress-related genes by specifically binding to their HSE cis-acting element in promoter regions.

## 4 Discussion

HSF is an important TF family in the plant kingdom that has been identified in *A. thaliana*, rice, tomato, corn, pepper, cabbage, and other plants. There are 21 HSF members in *A. thaliana*, which are divided into three subfamilies. *AtHSFA6b* in Class A not only participates in the high temperature stress response but also acts as a positive regulator of the stress response pathway mediated by the ABA signal, which significantly improves plant salt and drought tolerance (Huang et al., 2016). There are 25 HSF members in rice, and *OsHsfB2b* negatively regulates drought and salt tolerance in rice (Xiang et al., 2013). A total of 41 HSF members were identified from *Phyllostachys pubescens*, whose promoters contain many cis-acting elements related to stress. *PeHSFs* can be induced by gibberellin (GA) and naphthylacetic acid (NAA) and are highly expressed in panicles and young shoots, which may play a significant role in reproductive growth and organ development. As many as 49 HSF members were identified in tobacco, among which *NtHSF03* and *NtHSF12* were induced by high temperatures (Guo et al., 2022). *Zizania aquatica* has 28 HSF members, and 14 of them were highly expressed under high temperature stress (Cai et al., 2022).

In this study, we identified a total of thirty HSF members with conserved HSF domains from poplar, which all belong to acidic and hydrophilic proteins. All the PtHSFs were in nucleus, but PtHSF16 was also located in the whole cell, which was interesting. PtHSFs can be divided into three subfamilies (Classes A–C) (Huang et al., 2016). The structure of PtHSFs in the same subfamily is similar, while there are some differences among individual members. For example, PtHSF14 (Potri.006G226800.1) and PtHSF30 (Potri.T137400.1) have two intron structures, while the others contain one intron. Therefore, PtHSF14 and PtHSF30 may have experienced the cutting or insertion of gene fragments in the process of evolution (Staiger and Brown, 2013; Li et al., 2016). In addition to the common HSF conserved domain, PtHSF04 also contains a unique DivlC superfamily domain, which is a sporangium-forming domain of *Bacillus subtilis*. The SMC_prok_B domain represents an important domain for the maintenance of chromosome structure in *B. subtilis* (Soppa et al., 2002), and has an obvious inhibitory effect on plant pathogenic bacteria (Moo-Koh et al., 2022), which are present in PtHSF02, PtHSF07, and PtHSF12. In addition, all PtHSF members contain motifs 1–3, which may be the core components of the HSF domain. Class A had the largest number of PtHSF members with the largest number of motifs and the most complex conserved domain, which may have evolved from other functions.

Gene replication represents a general process of species evolution that can produce new functional genes and promote species evolution (Lynch and Conery, 2000). There are three modes of conventional gene replication: segmental replication, tandem repetition, and translocation events (Cannon et al., 2004). Rice has nine fragment repeat pairs in the HSF family (Guo et al., 2008), and *P. pubescens* has two tandem repeat pairs and twenty-seven fragment repeat pairs (Huang et al., 2021). In the study, we detected eleven fragment repeat pairs in PtHSFs, so segmental repetition dominates the expansion of the HSF family in poplar. Ka/Ks values of poplar HSF fragment repeats were all far less than 1, indicating these homologous genes underwent synonym mutation that was subjected to not only natural selection but also strong purification selection. In addition, every HSF fragment duplication gene belongs to the same subfamily in poplar, which may perform similar functions. There was a significant collinearity of HSF genes between poplar and *Arabidopsis*, like *PtHSF17* (*Potri.008G157600.1*)/*AtHSF6b* (*At3G22830.1*) and *PtHSF19* (*Potri.010G08200.1*)/*AtHSF6b* (*At5G43840.1*) (**Figure 4B**; **Supplementary Table S6**). Studies have shown that *AtHSF6b* mediates the ABA pathway to negatively regulate the drought resistance of *A. thaliana* (Wenjing et al., 2020), so it can be inferred that *PtHSF17* and *PtHSF19* may also be related to drought stress. In addition, *PtHSF19* also corresponds to three rice HSF genes (*LOC_Os03g06630.1*, *LOC_Os03G53340.1*, and *LOC_Os10G28340.1*), which are sensitive to high temperature, cold, and oxidative stress (Mittal et al., 2009; Mittal et al., 2012). The direct homologous gene of *PtHSF01* (*Potri.001G1081.1*) is *OsHsfB2b* (*LOC_Os08g43334.1*), which negatively regulates drought and salt tolerance in rice (Xiang et al., 2013). The direct homologous gene of *PtHSF09* (*Potri.004G062300.1*) is *OsHSF9* (*LOC_Os01g54550.1*), which is sensitive to temperature and improves cadmium resistance in rice (Shim et al., 2009). *OsHsfA9* (*LOC_Os03G12370.1*) is the direct homologous gene of *PtHSF16* (*Potri.008G136800.2*) and *PtHSF20* (*Potri.010G104300.1*), which can not only promote reproductive development of plants but also improve stress resistance (Chauhan et al., 2011). Therefore, these homologous *PtHSFs* may carry out similar functions in stress responses.

The promoters of poplar HSF family members contain many elements with various functions. It can be inferred that these genes may be involved in multiple signaling pathways and carry out a critical role in regulating development, abiotic stress, and hormone synthesis in poplar. The HSF gene is well-known to respond to high temperature stress (Guo et al., 2016). In this study, we explored the biological function of poplar HSF family members under salt stress. Among the 30 HSF family members, 25 *PtHSFs* respond to salt stress in different tissues, with thirteen upregulated genes and fourteen downregulated genes. Among them, *PtHSF21* was upregulated 4.5 times, 5.5 times, and 4.2 times in roots, stems, and leaves, respectively. This gene may perform a significant role in responding to salt stress; it was selected for further function validation.

We cloned *PsnHSF21* from *P. simonii × P. nigra*, which belongs to the Class C subfamily in poplar and is accurately located in the nucleus. PsnHSF21 contains three α helical bundles and four inverted parallel β folds, which are important DBD structures in the HSF domain (Sangster and Queitsch, 2005). Among the 10 interacting proteins of PsnHSF21, Hsp81.4 (Potri.001G286700.1) and HSC70-5 (Potri.015G078000.1) have been proved to be significantly induced by heat stress, and HSC70-5 is involved in seed development. HSP90.7 (Potri.005G241100.1) can improve drought and salt tolerance in plants through the ABA pathway (Song et al., 2009). Therefore, PsnHSF21 may perform a similar function or cooperate with the above proteins to promote plant development and participate in the stress response.

In this study, we obtained seven transgenic poplar lines overexpressing *PsnHSF21* (OE). Under normal conditions, there was no obvious difference in morphology between OE and WT. Under salt stress, the growth state of OE was significantly better than WT. After external stimulation, plants will undergo a series of physiological changes, especially the accumulation of ROS dominated by hydrogen peroxide (H_2_O_2_), the hydroxyl radical (OH−), and the superoxide anion (Erpen et al., 2018). SOD and POD are antioxidant enzymes that efficiently scavenge reactive oxygen species (ROS) (Feng et al., 2016; Survila et al., 2016). In this study, POD activity in OE was higher than that in WT, and SOD and POD gene expressions in OE were also higher than those in WT. This indicated that OE had better ROS scavenging ability. The H_2_O_2_ content of OE was lower than that of WT after salt stress, and the OE leaves with weaker DAB staining also indicated that there was less H_2_O_2_ content in OE. Free proline is an important index to detect plant growth status and evaluate plant stress resistance (Abrahám et al., 2010). Electrical conductivity, MDA content, and Evans blue staining are also indicators to evaluate the degree of cell damage. After salt stress, the proline content of OE was higher than that of WT, while the electrical conductivity and MDA content of OE were lower than those in WT. It is well recognized that abscisic acid (ABA) is widely involved in the stress response network to various environmental and abiotic stresses like drought, salinity, osmotic stress, and low temperature (Hamisch et al., 2016; Yu et al., 2017). Hydroxyproline glycoprotein (HRG) can improve plant antibacterial and disease resistance (Showalter et al., 2016). Gibberellin (GA) is a key hormone involved in plant development and stress response (Liu et al., 2018). Interestingly, the expression of the above stress-resistant genes in OE was higher than that in WT. Overall, the above results illustrated that PsnHSF21 could improve the salt resistance of OE by regulating these stress-related genes.

PsnHSF21 had self-activating activity, and the function region was at the C-terminal 150–212 aa, indicating the gene could form a stable transcription initiation complex and exert protein efficiently (**Figure 11A**). In animals and plants, HSE elements can be specifically recognized and bound by helix-transduction-helix motifs in the DNA binding domain (DBD) of HSF (Harrison et al., 1994; Schultheiss et al., 1996). When plants are subjected to high temperatures or other stresses, HSFs form HSF active trisomies through binding to hydrophobic OD, which accurately recognizes and binds to HSE elements in the gene promoter of the heat shock protein HSP gene, molecular chaperone, or other downstream genes to activate their transcription (Döring et al., 2000). For example, CmHSFA4 with transcriptional activation activity was identified in *Chrysanthemum*, which could respond to salt stress in combination with HSE elements (Li et al., 2018). In the study, the PsnHSF21 protein was proved to specifically bind to HSE elements, which indicates PsnHSF21 may regulate the expression of downstream salt-resistant genes by binding to HSE elements in their promoters.

## 5 Conclusions

In this study, a total of 30 HSF family genes were identified in poplar, all of which had HSF conserved domains. We systematically analyzed the gene structure, chromosome distribution, promoter cis-acting elements, gene duplication, and gene evolution of 30 PtHSFs and explored their expression pattern under salt stress by RNA-Seq and RT-qPCR. Among them, we identified a significantly salt-induced and nucleus-localized gene, *PsnHSF21*. We obtained seven transgenic poplar lines overexpressing *PsnHSF21* (OE) by the leaf-disk method. OE displayed morphological and physiological advantages under salt stress, for example, higher plant height and root length, enhanced ROS scavenging ability, and reduced cell damage compared to WT. At the molecular level, the relative expression levels of stress-related genes such as ABA and GA were significantly higher in OE. Moreover, PsnHSF21 was proven to specifically bind to the stress-resistant element HSE, and the self-activated region was at the C-terminal 150–212 aa. In conclusion, *PsnHSF21* regulates stress-related genes by specifically binding to the HSE element in their promoter regions to improve salt tolerance in poplar. This study provides a basis for understanding the biological function of the HSF gene in poplar under salt stress.

## Data availability statement

The original contributions presented in the study are included in the article/**Supplementary material**. Further inquiries can be directed to the corresponding authors.

## Author contributions

TJ and GQ designed the research. QG and RW conducted the experiments, analyzed the data, and wrote the manuscript. MX, JJ, and XM performed the data analysis. WY revised the manuscript. All authors contributed to the article and approved the submitted version.
