# Supplementary material for: Genome-wide analysis of HSF family and overexpression of PsnHSF21 confers salt tolerance in Populus simonii × P. nigra
Source: Front Plant Sci. 2023 Apr 26;14:1160102. doi: 10.3389/fpls.2023.1160102 (PMC10187788; doi:10.3389/fpls.2023.1160102)
Supplement: Supplementary file 1 [file DataSheet_1.docx]

Supplementary Material

Genome-Wide Analysis of HSF Family and Over-Expression of *PsnHSF21* Confers Salt Tolerance in *Populus simonii × P.nigra*

**Qing Guo^1, 2, †^, Ran Wei^1,†^, Min Xu^1^, Wenjing Yao^1,3^, Jiahui Jiang^1^, Xujun Ma^1^, Guanzheng Qu^1,*^, Tingbo Jiang^1,*^**

^1^State Key Laboratory of Tree Genetics and Breeding, Northeast Forestry University, Harbin, China

^2^School of Architecture and Civil Engineer, Heilongjiang University of Science and Technology, Harbin, China

^3^Co-Innovation Center for Sustainable Forestry in Southern China/Bamboo Research Institute, Nanjing Forestry University, Nanjing, China

*** Correspondence:**Guanzheng Qu

gzqu@nefu.edu.cn

Tingbo Jiang

[tbjiang@nefu.edu.cn](mailto:tbjiang@nefu.edu.cn)

†These authors have contributed equally to this work

# Supplementary Sheet

**root**

|  | **Base Mean** | **log2 Fold Change** | **lfcSE** | **stat** | **pvalue** |  |
| --- | --- | --- | --- | --- | --- | --- |
| PTHSF02 | 189.4133312 | -1.589009671 | 0.513921331 | -3.091931729 | 0.001988586 | down |
| PTHSF03 | 22.83224729 | -6.049663021 | 1.128878152 | -5.359004433 | 8.36818E-08 | down |
| PTHSF04 | 193.5942872 | -1.980703422 | 0.290324112 | -6.822386918 | 8.95401E-12 | down |
| PTHSF06 | 24.14502746 | -5.537540045 | 1.041845533 | -5.315125775 | 1.06584E-07 | down |
| PTHSF07 | 59.05508854 | -2.123414753 | 0.376007154 | -5.647272217 | 1.63014E-08 | down |
| PTHSF18 | 22.62039793 | -5.440179067 | 0.958301703 | -5.676895963 | 1.37161E-08 | down |
| PTHSF20 | 46.32997172 | -1.741404661 | 0.391312447 | -4.450164244 | 8.58046E-06 | down |
| PTHSF22 | 400.7174757 | -1.398406822 | 0.301681804 | -4.635370133 | 3.56299E-06 | down |
| PTHSF24 | 95.76668937 | -2.561443992 | 0.350550407 | -7.306920606 | 2.73334E-13 | down |
| PTHSF25 | 50.05975242 | -7.196313216 | 1.210057997 | -5.947081239 | 2.72966E-09 | down |
| PTHSF27 | 17.39028444 | -1.634176863 | 0.692131852 | -2.361077386 | 0.018221928 | down |
| PTHSF05 | 101.7321586 | 2.372683287 | 0.408685799 | 5.80564163 | 6.412E-09 | up |
| PTHSF10 | 190.6866255 | 1.206158862 | 0.404787072 | 2.979736623 | 0.002884963 | up |
| PTHSF12 | 639.7623132 | 1.938847394 | 0.197701053 | 9.806965448 | 1.05081E-22 | up |
| PTHSF13 | 43.2831222 | 4.551202546 | 0.734409047 | 6.197094883 | 5.75148E-10 | up |
| PTHSF17 | 6.594500685 | 4.150985564 | 1.54115555 | 2.693424142 | 0.007072224 | up |
| PTHSF19 | 49.54095722 | 2.459930487 | 0.630611655 | 3.90086429 | 9.58499E-05 | up |
| PTHSF26 | 1016.719803 | 1.883052895 | 0.193622756 | 9.725369771 | 2.3505E-22 | up |
| PTHSF28 | 924.1471856 | 2.015288024 | 0.311843239 | 6.462503505 | 1.02985E-10 | up |
| PTHSF30 | 264.5986405 | 1.00788548 | 0.430329565 | 2.342124647 | 0.01917431 | up |

**stem**

|  | **Base Mean** | **log2 Fold Change** | **lfcSE** | **stat** | **pvalue** |  |
| --- | --- | --- | --- | --- | --- | --- |
| PTHSF03 | 23.98304577 | -3.298216357 | 0.640371739 | -5.15047145 | 2.59832E-07 | down |
| PTHSF05 | 43.20096686 | -2.399552276 | 0.614797245 | -3.902997767 | 9.50086E-05 | down |
| PTHSF06 | 85.13449886 | -2.037233228 | 0.625031447 | -3.259409165 | 0.001116445 | down |
| PTHSF10 | 54.1678984 | -1.156012228 | 0.472453972 | -2.446825079 | 0.014412077 | down |
| PTHSF12 | 65.0902456 | -1.631210604 | 0.336512408 | -4.84740106 | 1.25089E-06 | down |
| PTHSF18 | 3.486880982 | -3.916253932 | 1.924563389 | -2.03487916 | 0.041863033 | down |
| PTHSF25 | 61.44588977 | -2.948132308 | 0.416928002 | -7.071082528 | 1.5373E-12 | down |
| PTHSF27 | 30.31088626 | -1.5607753 | 0.763764418 | -2.043529736 | 0.041000027 | down |
| PTHSF21 | 105.940682 | 2.465265952 | 0.531366718 | 4.639481297 | 3.49285E-06 | up |
| PTHSF26 | 442.5459491 | 1.341248588 | 0.253470617 | 5.291534781 | 1.21294E-07 | up |

**leaf**

|  | **Base Mean** | **log2 Fold Change** | **lfcSE** | **stat** | **pvalue** |  |
| --- | --- | --- | --- | --- | --- | --- |
| PTHSF15 | 291.5323639 | -1.065190352 | 0.540030141 | -1.97246463 | 0.04855659 | down |
| PTHSF21 | 538.8354158 | -5.202308505 | 1.917507517 | -2.713057685 | 0.00666655 | down |
| PTHSF01 | 186.3880434 | 1.17263465 | 0.236288604 | 4.962721988 | 6.9512E-07 | up |
| PTHSF03 | 3.202329075 | 1.250790146 | 0.609260422 | 2.052964711 | 0.040076 | up |
| PTHSF05 | 14.63588686 | 2.198497242 | 0.377054026 | 5.830722094 | 5.5188E-09 | up |
| PTHSF11 | 11.18919826 | 1.672703283 | 0.747799965 | 2.236832523 | 0.025297288 | up |
| PTHSF28 | 20.84966867 | 2.000593934 | 0.304364032 | 6.573030069 | 4.93015E-11 | up |

# Supplementary Figures

## Supplementary Figure 1


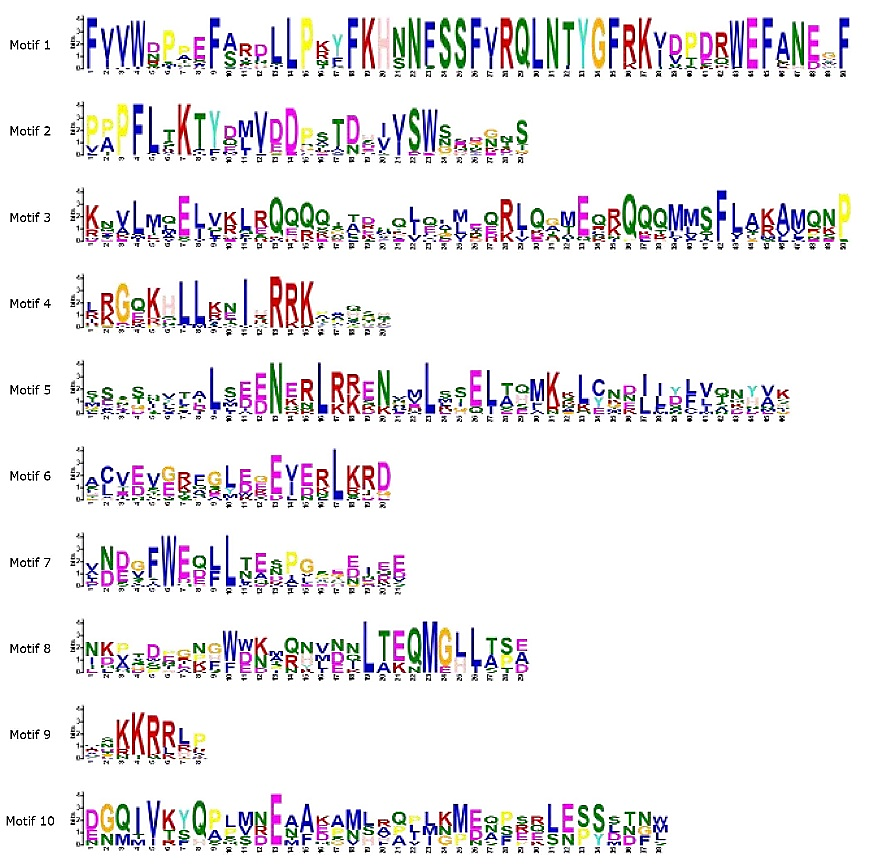


**Supplementary Figure 1.** The information of motif 1 to 10.

## Supplementary Figure 2


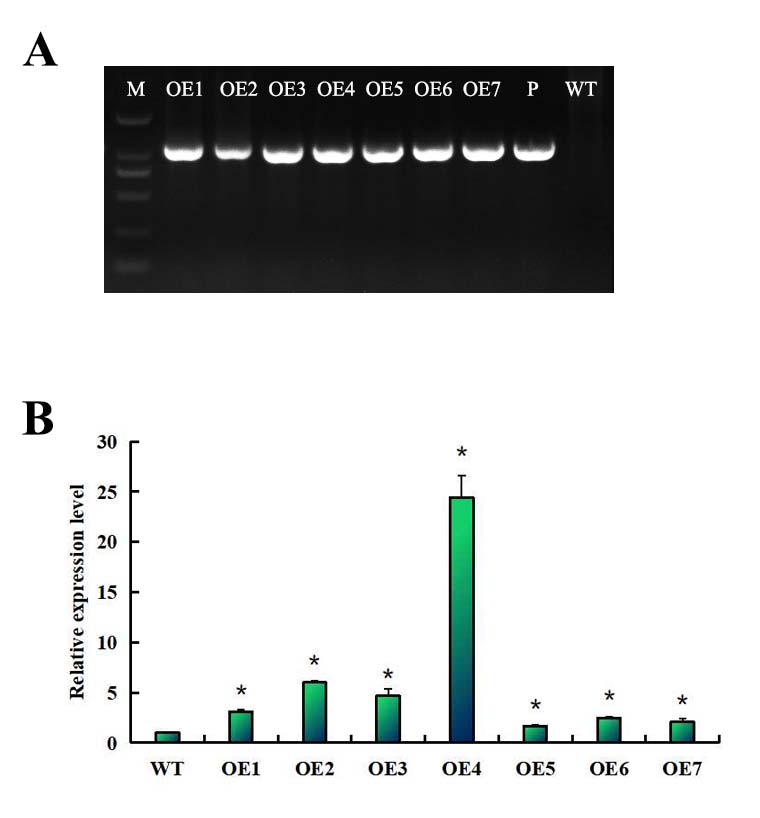


**Supplementary Figure 2.** (A) The PCR detection of the over-expressing PsnHSF21 poplar lines. (B) The detection of PsnHDZ01expression level in transgenic poplar.

## Supplementary Figure 3


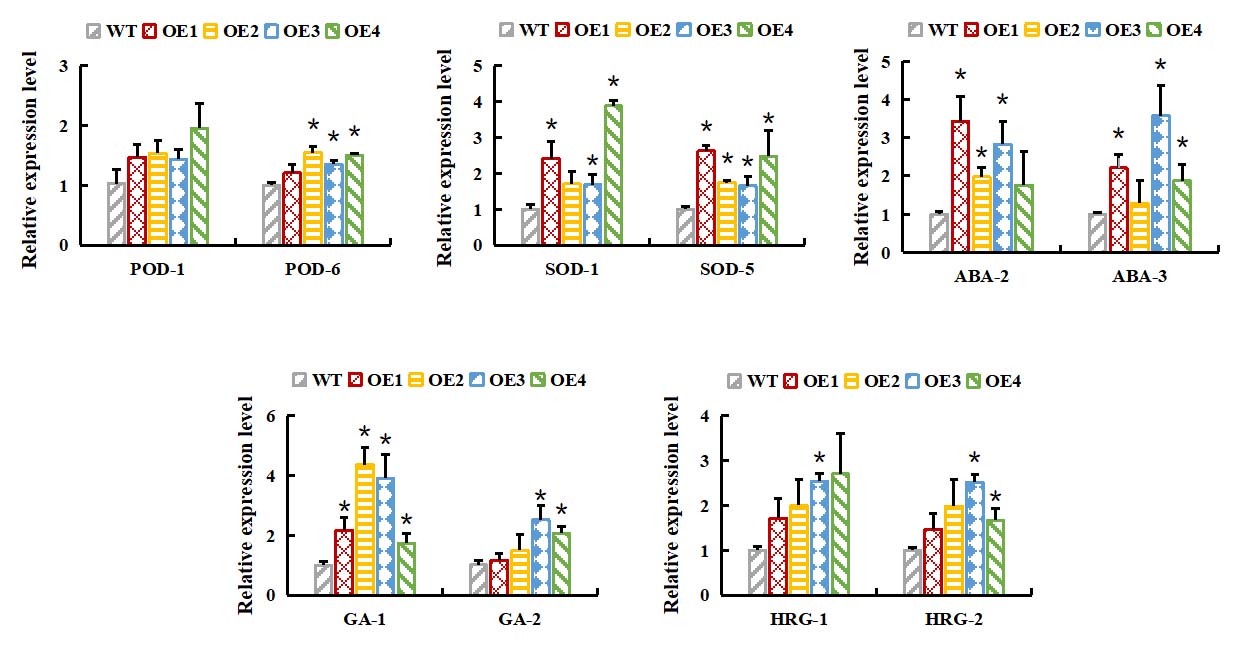


**Supplementary Figure 3.** RT-qPCR analysis showed that the relative expression levels of POD, SOD, ABA, GA, HRG genes in over-expressing PsnHSF21 poplar.

# Supplementary Tables

## Supplementary Table S1

**Supplementary Table S1. The primer sequences for RT-qPCR**

| **Gene Name** | **Gene ID** | **5’ primers** | **3’ primers** |
| --- | --- | --- | --- |
| *PsnHSF03* | *Potri.001G273700.1* | CATCCCAACACCATCACCAGCAC | GATCCTGACATGAAGAGCCCAG |
| *PsnHSF06* | *Potri.002G124800.1* | CCCAAGCAGAACCAGCATCTCTC | GCTGGGAGCAACAGGCTTGACATG |
| *PsnHSF18* | *Potri.004G042600.1* | CTGCCCTCTCAGAAGACAACCAG | GAACCCGGCTCTATAAGCTTGGG |
| *PsnHSF21* | *Potri.011G051600.1* | CCACCTTCCACGGTGCTAGTAG | CCAAGAACAAGAAGCCCTGCTCC |
| *PsnHSF25* | *Potri.014G027100.1* | CACCATCATCAGCACCACCCAC | CTCCACCACCAATAACTGTGGCG |
| *PsnHSF28* | *Potri.016G056500.1* | GGGGTCTTGAGCACTGAGCTTAC | CCTCGGCTTCCAATCTCACACC |
| *Act* | JM986590 | ACCCTCCAATCCAGACACTG | TTGCTGACCGTATGAGCAAG |

## Supplementary Table S2

**Supplementary Table S2. The primer sequences for *PsnHSF21* gene Cloning**

| **Gene Name** | **5’ primers** | **3’ primers** |
| --- | --- | --- |
| *PsnHSF21* | CATGGCGGCCACTTTCTCTCAGCTTG | CGCACACTAGAGAGAACTAG |

## Supplementary Table S3

**Supplementary Table S3. The primer sequences for GFP vector Construction**

| **Vector** | **5’ primers** | **3’ primers** |
| --- | --- | --- |
| PBI121 | CCATCGTTGAAGATGCCTCTGC | CTCTTCGCTATTACGCCAGCTG |
| PBI121-PsnHSF21*-*GFP | GCGTCGACGCATGCCTGCAGGTCGACGATTC | GACTAGTACATAGCCTCCTGGTTTTGTTC |

## Supplementary Table S4

**Supplementary Table S4 The primer sequences for RT-qPCR of stress-related genes**

| **Gene ID** | **5’ primers** | **3’ primers** |
| --- | --- | --- |
| SOD1 | GCCTTGCCTGAGATACTTAC | GCTTCAGTCATAGTCTTCAC |
| SOD5 | CTCTCATTCTCCTCTCCGTG | CCAGGCACACCACCAACTTG |
| POD1 | GTCTTATGCTGACTTCTACC | AATCCAGAACGCTCCTTGTG |
| POD6 | GTTCCATCTCTTACTTGTTC | CTTGTTGGTTGCAGTGGAGG |
| HRG1 | CGTCATCTCCTCCAGTTGCC | GAATGGAATAGAGGGAATAG |
| HRG2 | CACCTCCGCAACACTACCAC | GTTGCGGATACTGATGTTGG |
| ABA2 | GGTATGGCTCTATCTTCAAG | GAAGAGAGTCCTTGGCAATG |
| ABA3 | ATCTCTGGCAACTTCGCTCC | AGTGAACCTGCTAGAATAGC |
| GA1 | GAGAACATTGAGAGTGCTAG | CAAGAGGAGAGCCAACTATG |
| GA2 | AAGGCTGTTGTGGTGTCTGT | TTACCACCTGAACTATCGCC |

## Supplementary Table S5

**Supplementary Table S5 The primer sequences for yeast experimental vector Construction**

| **Vector** | **5’ primers** | **3’ primers** |
| --- | --- | --- |
| pGBKT7 | TAATACGACTCACTATAGGGCG | TTTTCGTTTTAAAACCTAAGAGTC |
| pAbAi | GTTCCTTATATGTAGCTTTCGACAT | GGCAGTTTGGAGGTCTCTCTGATAG |
| pGADT7 | CTATTCGATGATGAAGATACCCCACCAAACCC | GTGAACTTGCGGGGTTTTTCAGTATCTACGATT |
| BD-PsnHSF21 | GGAATTCCATATGGCATGCCTGCAGGTCGACGATTC | TCCCCCGGGACATAGCCTCCTGGTTTTGTTC |
| BD-PsnHSF21-1 | GGAATTCCATATGGCATGCCTGCAGGTCGACGATTC | TCCCCCGGGCTGACGGATGAAGCTAGAGAAG |
| BD-PsnHSF21-2 | GGAATTCCATATGCTTAACACTTATGGATTCAAG | TCCCCCGGGACATAGCCTCCTGGTTTTGTTC |
| BD-PsnHSF21-3 | GGAATTCCATATGGCATGCCTGCAGGTCGACGATTC | TCCCCCGGGCTCTTGATTAGAAGAAGCCT |
| BD-PsnHSF21-4 | GGAATTCCATATGAATGCAACAATTGACATGGAAG | TCCCCCGGGACATAGCCTCCTGGTTTTGTTC |
| AB-HSE | AGCTTGAATTCGAATTCGAATTCGAGCT | CGAATTCGAATTCGAATTCA |
| AD-PsnHSF21 | GGAATTCCATATGGCATGCCTGCAGGTCGACGATTC | CGCGGATCCGACATAGCCTCCTGGTTTTGTTC |

## Supplementary Table S6

**Supplementary Table S6 The relationship collinearity for paralogous gene pairs**

| **No.** | **Gene ID (X)** | **Gene X** | **Chr X** | **Gene Y** | **chr Y** | **Relationship** |
| --- | --- | --- | --- | --- | --- | --- |
| 1 | *PtHSF01* | *Potri.001G108100.1* | Chr01 | *LOC_Os04g48030.1* | chr04 | Conllinearity |
| 2 | *PtHSF01* | *Potri.001G108100.1* | Chr01 | *LOC_Os08g43334.1* | chr08 | Conllinearity |
| 3 | *PtHSF01* | *Potri.001G108100.1* | Chr01 | *LOC_Os09g35790.1* | chr09 | Conllinearity |
| 4 | *PtHSF05* | *Potri.002G048200.1* | Chr02 | *LOC_Os03g06630.1* | chr03 | Conllinearity |
| 5 | *PtHSF05* | *Potri.002G048200.1* | Chr02 | *LOC_Os03g58160.1* | chr03 | Conllinearity |
| 6 | *PtHSF05* | *Potri.002G048200.1* | Chr02 | *LOC_Os07g08140.1* | chr07 | Conllinearity |
| 7 | *PtHSF06* | *Potri.002G124800.1* | Chr02 | *LOC_Os08g36700.1* | chr08 | Conllinearity |
| 8 | *PtHSF06* | *Potri.002G124800.1* | Chr02 | *LOC_Os09g28200.1* | chr09 | Conllinearity |
| 9 | *PtHSF09* | *Potri.004G062300.1* | Chr04 | *LOC_Os01g54550.1* | chr01 | Conllinearity |
| 10 | *PtHSF16* | *Potri.008G136800.2* | Chr08 | *LOC_Os03g12370.1* | chr03 | Conllinearity |
| 11 | *PtHSF17* | *Potri.008G157600.1* | Chr08 | *LOC_Os03g06630.1* | chr03 | Conllinearity |
| 12 | *PtHSF17* | *Potri.008G157600.1* | Chr08 | *LOC_Os03g53340.1* | chr03 | Conllinearity |
| 13 | *PtHSF17* | *Potri.008G157600.1* | Chr08 | *LOC_Os07g08140.1* | chr07 | Conllinearity |
| 14 | *PtHSF17* | *Potri.008G157600.1* | Chr08 | *LOC_Os10g28340.1* | chr10 | Conllinearity |
| 15 | *PtHSF19* | *Potri.010G082000.1* | Chr10 | *LOC_Os03g06630.1* | chr03 | Conllinearity |
| 16 | *PtHSF19* | *Potri.010G082000.1* | Chr10 | *LOC_Os03g53340.1* | chr03 | Conllinearity |
| 17 | *PtHSF19* | *Potri.010G082000.1* | Chr10 | *LOC_Os10g28340.1* | chr10 | Conllinearity |
| 18 | *PtHSF20* | *Potri.010G104300.1* | Chr10 | *LOC_Os03g12370.1* | chr03 | Conllinearity |
| 19 | *PtHSF25* | *Potri.014G027100.1* | Chr14 | *LOC_Os08g36700.1* | chr08 | Conllinearity |
| 20 | *PtHSF25* | *Potri.014G027100.1* | Chr14 | *LOC_Os09g28200.1* | chr09 | Conllinearity |
| 21 | *PtHSF17* | *Potri.008G157600.1* | Chr08 | *AT3G22830.1* | LG03 | Conllinearity |
| 22 | *PtHSF19* | *Potri.010G082000.1* | Chr10 | *AT3G22830.1* | LG03 | Conllinearity |
| 23 | *PtHSF19* | *Potri.010G082000.1* | Chr10 | *AT5G43840.1* | LG05 | Conllinearity |
